# Cysteine residues contribute to the regulation of *Arabidopsis* state transition 7 kinase

**DOI:** 10.1002/1873-3468.15032

**Published:** 2024-10-11

**Authors:** Iskander M. Ibrahim, Ji H. Lee, Seth Weaver, Ronard Kwizera, Jeremy R. Lohman, Sujith Puthiyaveetil

**Affiliations:** ^1^ Department of Biochemistry and Center for Plant Biology Purdue University West Lafayette IN USA; ^2^ Present address: Department of Biological Sciences Towson University Towson MD USA; ^3^ Present address: Department of Molecular Genetics and Microbiology Duke University Medical Center Durham NC USA; ^4^ Present address: Department of Biochemistry and Molecular Biology Michigan State University East Lansing MI USA

**Keywords:** LHCII, PQ pool, state transitions, STN7/STT7, thiol regulation, thioredoxin

## Abstract

State transitions are an acclimatory response by which plants, algae, and cyanobacteria counteract photosynthetic inefficiency caused by changes in incident light quality. In plants and green algae, state transition 7 (STN7/STT7) kinase promotes state 2 transition. Conserved cysteine residues are implicated in STN7/STT7 regulation, but the precise nature of their involvement remains unclear. Here, an analysis of the STN7 thiols *in vitro* and a determination of their midpoint redox potential indicate that the lumenal disulfide linkage is unlikely to be redox regulated while the stromal cysteines form a regulatory intramolecular disulfide. We further show that thioredoxin *f*1 (Trx‐*f*1) reduces the STN7 stromal disulfide linkage as consistent with a Trx‐*f*1‐mediated inhibition of the kinase under high light.

## Graphical abstract

This study reveals how two intramolecular disulfide linkages regulate the activity of state transition 7 (STN7) kinase, which controls photosynthetic light harvesting in plants and algae. The lumenal disulfide bond may facilitate STN7's interaction with Rieske protein for kinase activation while the stromal disulfide bond is reduced by thioredoxin *f*1 (Trx‐*f*1) to inhibit the kinase in high light.

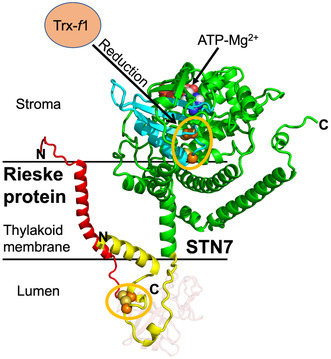

## Abbreviations

**AA**, amino acid

**Cyt *b***_**6**_ ***f***, cytochrome *b*_6_ *f*

**DTT**, dithiothreitol

**EC**_**50**_, half maximal effective concentration

***E***_**h**_, redox potential

***E***_**m7**_, midpoint potential at pH 7

**H**_**2**_ **O**_**2**_, hydrogen peroxide

**IPTG**, isopropyl β‐d‐1‐thiogalactopyranoside

**LB**, lysogeny broth

**LHCII**, light harvesting complex II

**LTO1**, thylakoid lumen thiol oxidoreductase 1

**MBP**, maltose‐binding protein

**mPEG‐MAL**, methoxypolyethylene glycol maleimide

**PAGE**, polyacrylamide gel electrophoresis

**PPH1**, protein phosphatase 1

**PQ**, plastoquinone

**PSI**, photosystem I

**PSII**, photosystem II

**Q**_**o**_ **‐site**, quinol oxidation site

**SDS**, sodium dodecyl sulfate

**STN7/STT7**, state transition 7

**TAP38**, thylakoid associated phosphatase of 38 kDa

**Trx**, thioredoxin

In oxygenic photosynthesis, solar energy is captured by two photosystems—photosystem II (PSII) and photosystem I (PSI). PSII and PSI are connected in series by a cytochrome *b*_6_ *f* (cyt *b*_6_ *f*) complex for linear electron transport from water to NADP^+^. Because of the distinct pigment and protein composition, the light‐harvesting systems of PSII and PSI absorb light at slightly different wavelengths [1]. Linear electron transport is optimal when the rate of solar energy conversion is equal at both photosystems. The spectral quality of sunlight in nature, however, fluctuates on a time scale of minutes to hours, causing uneven excitation of the two photosystems. State transitions were first described in red and green algae as a short‐term acclimatory response to unequal excitation of photosystems [2, 3]. Preferential excitation of PSII by a short wavelength light (light 2) induces state 2, wherein some of the absorbed light energy is redistributed to PSI at the expense of PSII. Conversely, long wavelength illumination (light 1), which preferentially excites PSI over PSII, initiates state 1. In state 1, some of the absorbed excitation energy is diverted to PSII to correct the imbalance caused by overexcitation of PSI. The transitions between state 1 and state 2 are known as state transitions [4, 5]. In plants and green algae, state transitions employ a pool of mobile light harvesting complex II (LHCII) peripheral antenna, which becomes redistributed to the rate‐limiting photosystem. State transition 7 (STN7/STT7) kinase [6, 7] phosphorylates LHCII and thus drives transition to state 2 while thylakoid associated phosphatase of 38 kDa/protein phosphatase 1 (TAP38/PPH1) [8, 9] dephosphorylates LHCII to promote state 1.

A key facet of the LHCII kinase regulation became apparent when it was discovered that an overreduced plastoquinone (PQ) pool activates the kinase under PSII‐specific low light condition [10]. Since then, it has been demonstrated that this activation requires the cyt *b*_6_ *f* complex [11, 12, 13], specifically a PQH_2_ molecule bound at the lumen‐side quinone‐binding pocket of cyt *b*_6_ *f* (Q_o_‐site) [14, 15]. How the Q_o_‐site‐bound quinol orchestrates the transmembrane activation of the stroma‐located kinase domain of STN7, however, remains an unsolved puzzle. STN7/STT7 interacts with the cyt *b*_6_ *f* complex, specifically with its Rieske iron sulfur protein subunit and a stromal loop of subunit IV, with implications for its activity [16, 17, 18, 19]. An early model invoked conformational change in cyt *b*_6_ *f* as important for activation of the kinase [20]. This conformational change in cyt *b*_6_ *f* is brought about by the movement of the Rieske iron sulfur protein subunit upon binding of the quinol in the Q_o_‐site. A variant of the conformational change model posits a role for the cyt *b*_6_ *f*‐bound chl *a* molecule in conveying the occupancy of the Q_o_‐site or the position of the Rieske Fe‐S center to the kinase [21, 22]. A separate thiol‐based activation mechanism has also been proposed when it was evident that STN7/STT7 contains a pair of conserved closely spaced cysteine residues (CxxxxC) in its thylakoid lumen‐located N‐terminal segment [23]. Removal of either or both cysteines renders the kinase inactive and impairs state transitions [16], suggesting an essential role for the lumenal dithiol in the functioning of STN7. A direct reduction of the STN7 lumenal disulfide bond by PQH_2_ or by the superoxide produced at the Q_o_‐site during quinol oxidation has been proposed to activate the kinase [18, 23]. As an activation mechanism, a transient dimerization of the kinase via intermolecular lumenal disulfide linkages has also been discussed [17]. Furthermore, thylakoid lumen thiol oxidoreductase 1 (LTO1) has recently been shown to be required for STN7 activity by maintaining the lumenal dithiol of STN7 in an oxidized disulfide state [24]. It is thus important to characterize the redox behavior of STN7's lumenal cysteines.

A marked decrease in LHCII phosphorylation occurs at higher irradiances as consistent with an inhibition of LHCII kinase activity in high light [25]. Purified thioredoxins inhibit LHCII phosphorylation *in vitro*, raising the possibility that it is thioredoxin that inhibits STN7 in high light [26]. The identification of a conserved CxxxC motif in the stromal kinase domain of STN7 is consistent with this idea [23]. An analysis by Wunder *et al*. [27] interestingly found that lumenal cysteine mutants still interacted with thioredoxins, further supporting the notion that stromal cysteines are the real target sites of thioredoxin in STN7. This study also showed that STN7 interacted with Trx‐*f*1 rather than Trx‐*m*1 [27], in agreement with the Rintamäki *et al*.'s [26] original demonstration that Trx‐*f* was more effective than Trx‐*m* at inhibiting LHCII phosphorylation *in vitro*. However, an overexpression of thioredoxin *m*1 (Trx‐*m*1) in tobacco inhibited LHCII phosphorylation and prevented state transitions [28]. Whether this result, obtained with the overexpressed Trx‐*m*1 in tobacco, was a nonspecific effect of overexpression remains unclear. Regardless, evidence for a direct reduction of STN7 stromal cysteines by Trx‐*f*1 or other thioredoxins is currently lacking. In this study, we employ *in vitro* biochemical approaches to decipher the role of the conserved lumenal and stromal cysteine residues in regulation of STN7.

## Materials and methods

### Construction of recombinant plasmids

Coding sequences corresponding to *Arabidopsis thaliana* STN7 (*AT1G68830*) lumenal segment (AA 46–89), STN7 stromal segment (AA 162–216), mature Trx‐*m*1 (*AT1G03680*) (AA 71–179), mature Trx‐*m*2 (*AT4G03520*) (AA 76–112), mature Trx‐*f*1 (*At3g02730*) (AA 58–179), and LTO1 (*AT4G35760*) lumenal segment (AA 251–376) were amplified from cDNA using primer pairs listed in Table 1. PCR products were cut with *KpnI* and *XhoI* endonucleases (New England BioLabs, Ipswich, MA, USA) and cloned into pETG‐41A plasmid.

**Table 1:** Primer pairs used for cloning *STN7*, *Trx‐f*1, *Trx‐m1*, *Trx‐m2*, and *LTO1*. Sequences in lowercase are restriction site overhangs. Codons underlined are codons that are substituted from cysteine to serine.

Stn7‐Lum‐KpnI_F: GCGCGCggtaccGCTCAATTGATCGATA Stn7‐Lum‐XhoI_R: GCGGCGctcgagTCAAGCAGTGATCGTT
Stn7‐Stromal‐KpnI_F: GCGCGCggtaccGCGACTGAGTATGGTGCG Stn7‐Stromal‐XhoI_R: GCGGCGctcgagTCACTGCATCAAACCAGCAAG
Trx‐*f*1‐M‐KpnI_F: GCGCggtaccTGTAGCTTAGAAACCGTT Trx‐*f*1‐M‐XhoI_R: GCGCctcgagTCATCCGGAAGCAGCAGA
Trx‐*m*1‐M‐KpnI_F: GCGCggtaccGACACTGCTACAGGAATT Trx‐*m*1‐M‐XhoI_R: GCGCctcgagTTACAAGAATTTGTTGAT
Trx‐*m2*‐M‐KpnI_F: GCGCggtaccCAGGAAACTACTACCGAT Trx‐m2‐M‐XhoI_R: GCGCctcgagTCATGGCAAGAACTTGTC
LTO1‐Redox‐KpnI_F: GCGCGCggtaccTCACGCTCTGGTGACATT LTO1‐Redox‐XhoI_R: GCGGCGctcgagTTACTGAAGTTGATTGGT
Stn7‐Lum^C65S^_F: GGACTTCCATCTACGGTTATG Stn7‐Lum^C65S^_R: CATAACCGTAGATGGAAGTCC
Stn7‐Lum^C70S^_F: GTTATGGAGTCTGGTGATATG Stn7‐Lum^C70S^_R: CATATCACCAGACTCCATAAC

### Site‐directed mutagenesis

Mutagenesis of the conserved cysteine residues of the lumenal segment of STN7 (C65 and C70) to serine was made using the Phusion Site‐Directed Mutagenesis Kit (Thermo Fisher Scientific, Waltham, MA, USA). The primer pairs used are listed in Table 1. Mutagenesis was confirmed by sequencing (results not shown).

### Expression and purification of recombinant proteins

The recombinant plasmid constructs were introduced into BL21(DE3) chemically competent cells (New England BioLabs, Ipswich, MA, USA) by transformation. Colonies that grew on agar selection plates were used to inoculate overnight starter cultures in 10 mL lysogeny broth (LB) supplemented with 100 μg·mL^−1^ ampicillin. The overnight cultures were diluted to 1 : 100 in 1 L LB medium and grown at 37 °C until an optical density at 600 nm of 0.55 was reached. Recombinant protein overexpression was initiated by adding isopropyl β‐d‐1‐thiogalactopyranoside (IPTG) at a final concentration of 0.5 mm. The cultures were subsequently grown at 16 °C for a further 16 h. Cells were harvested by centrifugation at 9000 ***g*** for 10 min and the pellets were resuspended in lysis buffer (20 mm Tris/HCl pH 8, 300 mm NaCl, 25 mm imidazole, and 1 mm PMSF) and lysed with an EmulsiFlex‐C3 homogenizer (Avestin, Ottawa, Ontario, Canada). The lysate was separated by centrifugation at 39 000 ***g*** for 20 min at 4 °C. The supernatant was applied to a Ni^2+^ affinity column (Cytiva, Uppsala, Sweden) and the recombinant proteins purified according to the manufacturer's instructions.

### PEGylation

Free thiols on the STN7 lumenal segment were tagged with methoxypolyethylene glycol maleimide (mPEG‐MAL), MW = 5000. The purified recombinant STN7 lumenal segment was exchanged into a buffer containing 100 mm NaCl and 20 mm Tris/HCl pH 8.0 using PD MiniTrap G25 column (Cytiva). Aliquots of 15 μm final protein concentration were incubated with PEGylation buffer (100 mm NaCl, 20 mm Tris/HCl pH 8.0, 1 mm EDTA, and 1 mm mPEG‐MAL) at 25 °C for 1 h. Reactions were quenched by adding 5× SDS‐PAGE sample buffer and resolved on nonreducing 8% SDS‐urea‐PAGE gel.

### Redox treatment

Aliquots of 5 μm desalted recombinant WT and cysteine mutant lumenal segments of *A. thaliana* STN7 were incubated with a final concentration of 2 mm cysteine‐specific oxidant diamide, H_2_O_2_, or with the reductant dithiothreitol (DTT) for 30 min at room temperature. The reaction products were immediately resolved on a nonreducing 8% SDS‐urea‐PAGE gel.

### Redox titration

The purified STN7 lumenal and stromal segment proteins were buffer exchanged into a redox titration buffer (100 mm NaCl, 100 mm HEPES pH 7.0 or pH 8.0) with a MiniTrap G25 column. Aliquots of 5 μm STN7^C65S^ and STN7^C70S^ lumenal or stromal segments were equilibrated for 2 h at redox potentials ranging from −220 to −434 mV in the redox titration buffer at 22 °C. The different redox potentials were achieved by mixing different ratios of oxidized and reduced DTT at a final concentration of 2 mm. The redox‐titrated proteins were resolved on a nonreducing SDS‐urea‐PAGE gel as before. The oxidized and reduced proteins migrated differently on the gel and their band intensity, as quantified by the image lab software (Bio‐Rad, Hercules, CA, USA), was used as a reporter for the redox titration. The equilibrium redox potential (*E*_h_) was calculated by the Nernst equation:

$$E_{h}=E_{0}+29.6\;\log\frac{\left[{\mathrm{DTT}}_{\mathrm{ox}}\right]}{\left[{\mathrm{DTT}}_{\mathrm{Red}}\right]}$$

where [DTT_ox_] and [DTT_red_] are molar concentrations of oxidized and reduced DTT, respectively, and *E*_0_ is the standard redox potential of DTT at pH 7.0 of −327 mV and at pH 8.0 of −386 mV with adjustment of −59 mV per pH unit [29]. For the lumenal segment, the dithiol was calculated as maximum disulfide minus the disulfide at any given redox potential (*E*_h_). The dithiol was then expressed as the % maximum at that *E*_h_. This is to account for the fact that not all STN7 lumenal segment proteins can be oxidized in our experiment. The midpoint potential (*E*_m_) was determined by fitting the data using the prism software (GraphPad, Boston, MA, USA) with a nonlinear regression model:

$$Y=\frac{\text{Bottom}+\left(\mathrm{Top}-\text{Bottom}\right)}{\left(1+10^{\left(\mathrm{Log}{\mathrm{EC}}_{50}-X\right)\times \text{HillSlope}}\right)}$$

where *X* is the measured value and the Hill Slope of the straight line defining the tangent at the inflection point. The half maximal effective concentration (EC_50_) was calculated from the regression model.

## Results

### STN7 lumenal cysteines show less propensity to form intermolecular disulfide linkages

**Fig. 1:**
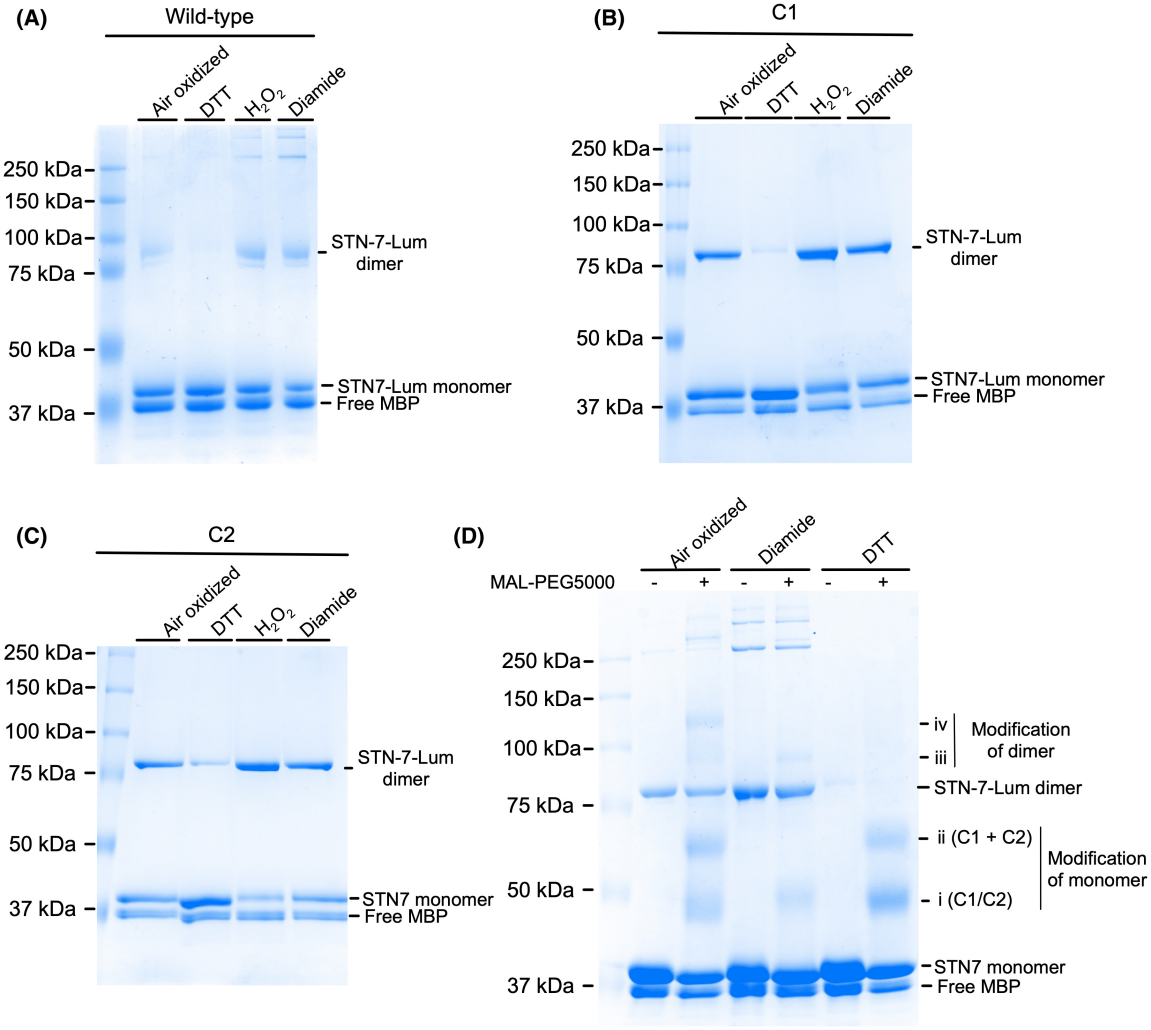
Effect of different redox agents on the oligomeric state of the lumenal segment of STN7. (A–C) redox‐treated wild‐type and mutant proteins as resolved on nonreducing SDS‐urea‐PAGE gel. (D) PEGylation of free cysteines in oxidized and reduced STN7 proteins.

To examine the *in vitro* biochemical behavior of STN7 lumenal cysteines, we overexpressed and purified the lumenal segment of *Arabidopsis* STN7. The lumenal segment, containing the two conserved cysteines (C65 and C70), was overexpressed as a fusion protein with an N‐terminal maltose‐binding protein (MBP) in *Escherichia coli*. Since MBP is devoid of cysteine residues, the observed thiol redox chemistry can arise only from the STN7 lumenal segment. Figure 1A shows the effect of different redox agents on the oligomeric state of the MBP‐tagged lumenal segment of STN7 on a nonreducing 8% SDS‐urea‐PAGE gel. Most of the air‐oxidized wild type recombinant STN7 migrates as a monomer. However, a small proportion of STN7 forms dimers consistent with previous *in vitro* observation of recombinant STN7 and of STN7 overexpression lines carrying a single Cys to Ser site‐directed mutation of either lumenal cysteines [24, 30]. In the presence of oxidizing agents H_2_O_2_ and diamide, a thiol‐specific oxidant, the proportion of dimers increases along with the appearance of higher‐order oligomers.

To determine how the mutagenesis of lumenal cysteines affects STN7 oligomerization, we replaced each cysteine with serine and resolved the mutant proteins on a nonreducing gel. Figure 1B,C shows STN7^C65S^ and STN7^C70S^ mutant proteins under various redox conditions. In the absence of either of the two cysteines, nearly half of the STN7 proteins form an intermolecular disulfide bond, with the proportion of dimers further increased with the addition of oxidizing agents. To further examine the redox state of STN7 lumenal thiols, we checked the presence of a free thiol group in the air‐oxidized wild type protein by covalently tagging the free thiol groups with the high molecular weight compound mPEG‐MAL (5 kDa). The oxidized disulfide‐bonded thiols in a protein are unavailable for PEGylation. PEGylation produced four distinct complexes for the air‐oxidized protein (lane 3, Fig. 1D). Complexes i and ii correspond to PEGylation of a single cysteine (C1 or C2) and both cysteines (C1 and C2) of the monomeric protein, respectively. The iii and iv complexes, with higher apparent molecular weights than the dimeric STN7, correspond to PEGylation of a single (C1 or C2) or both (C1C1 or C2C2) cysteine residues of the dimeric protein formed via a single intermolecular disulfide bond. The PEGylation of the dimeric air‐oxidized protein indicates that only one of the two conserved cysteines is involved in disulfide‐mediated dimerization of the STN7 *in vitro*. In the diamide‐oxidized protein, complexes ii and iv are absent, indicating that non‐PEGylated dimers formed under this condition is due to both conserved cysteines forming intermolecular disulfide bonds. Reducing the air‐oxidized and PEGylated STN7 resulted in the disappearance of complex iii and iv along with the dimers (lane 7, Fig. 1D). The reduced protein is thus made up of complex i and ii together with non‐PEGylated monomers. It is unclear why the oxidized intramolecularly disulfide‐bonded STN7 monomer is not apparent in our nonreducing urea gel (Fig. 1) or that of other's [24]. It is possible that oxidized (intramolecularly disulfide‐bonded) and reduced lumenal dithiol forms of STN7 cannot be separated on the gel as the shift in migration of the oxidized protein is too small to be detected or that the oxidized protein comigrates with a truncation product below the reduced monomer band in our gel (Fig. 1D).

### Midpoint redox potential of the lumenal cysteines of STN7

**Fig. 2:**
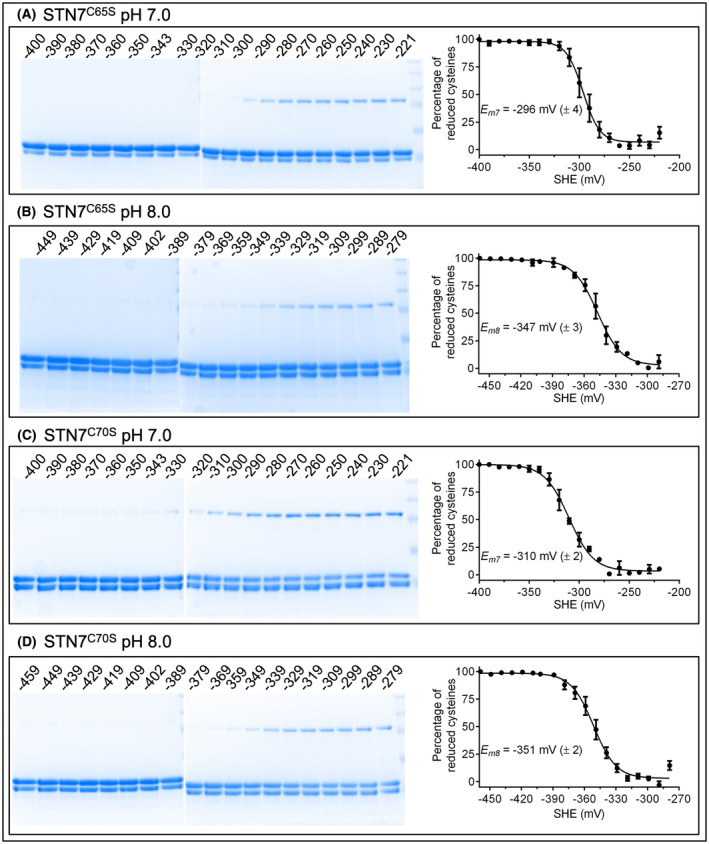
Redox titration of STN7^C65S^ and STN7^C70S^ mutant lumenal segment proteins. A and C are redox titration at pH 7.0 for STN7^C65S^ and STN7^C70S^, respectively; B and D are redox titration at pH 8.0 for STN7^C65S^ and STN7^C70S^, respectively. The corresponding nonreducing gel and redox plot are shown. The redox potential is indicated on top of each lane. For the redox plots, the y‐axis values correspond to percentage of maximally reduced cysteines. The solid line is the best fit to the Nernst equation. The data fit a two electrons slope (*n* = 2). The midpoint potential (*E*_m_) is indicated. Error bars represent standard error of mean (SEM).

To further characterize the redox properties of the lumenal cysteines, we determined midpoint redox potential of their intermolecular disulfide linkages by a redox titration. We used cysteine to serine mutants STN7^C65S^ and STN7^C70S^ for the titration to determine the *E*_m_ of each intermolecular disulfide. Titrations were performed in at least duplicate for both mutants at pH 7 (Fig. 2A–C) and pH 8 (Fig. 2B–D and Data S1). The titration yielded an average *E*_m_ value at pH 7 of −296 ± 4 mV for STN7^C65S^ mutant and −310 ± 2 mV for STN7^C70S^ mutant. At pH 8, an *E*_m_ value of −347 ± 3 mV for STN7^C65S^ mutant and −351 ± 2 mV for STN7^C70S^ mutant was apparent. Because redox titration of STN7^C65S^ mutant informs on the *E*_m_ value of C70, and vice versa for STN7^C70S^ mutant, our analysis indicates that C70 has a slightly more positive *E*_m_ than C65.

### LTO1 cannot oxidize lumenal dithiol into intermolecular disulfide linkages

**Fig. 3:**
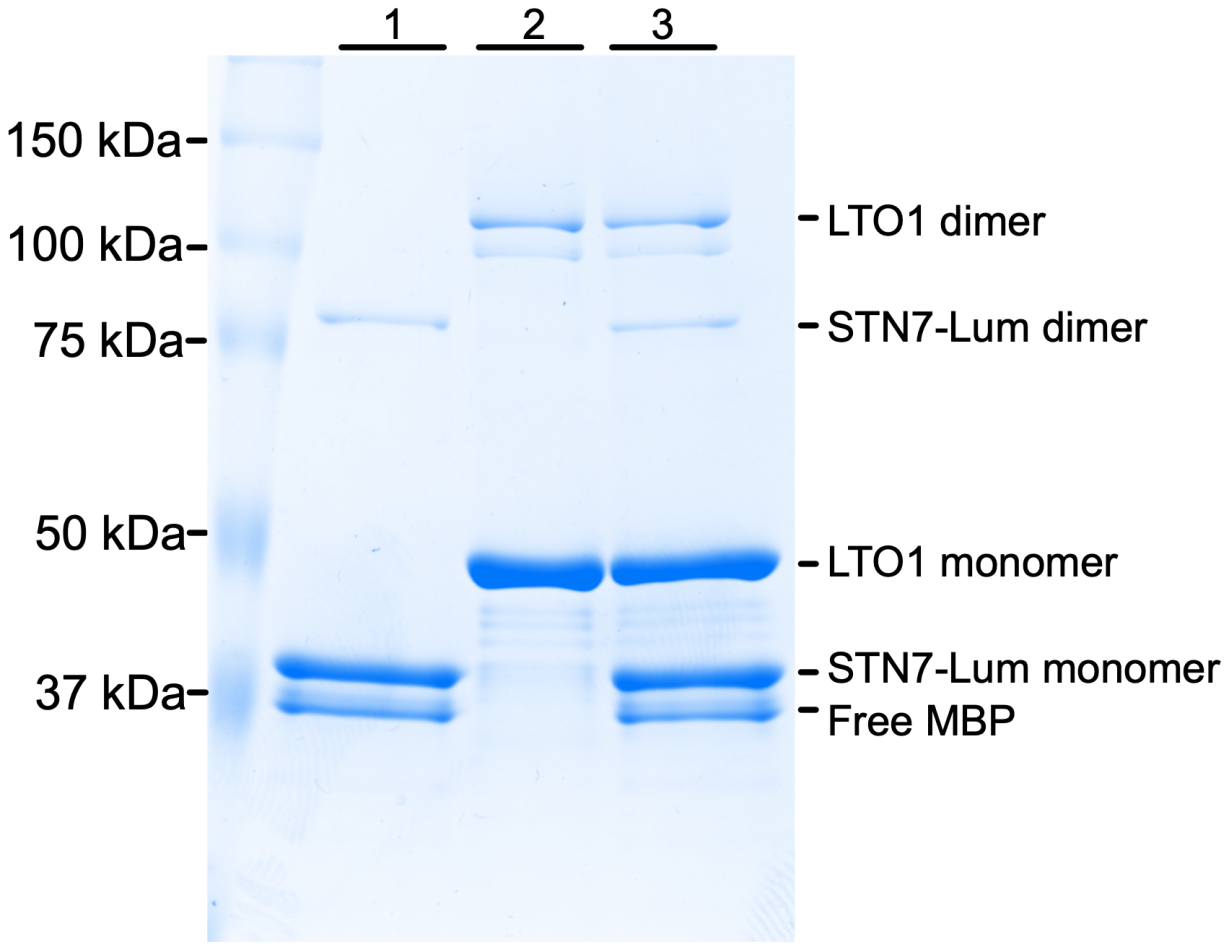
The effect of oxidized LTO1 on dimerization of STN7 lumenal segment. Lane 1 corresponds to the reduced STN7 protein after buffer exchange into a DTT‐free buffer; lane 2, the air‐oxidized LTO1 protein; and lane 3, the reduced STN7 protein after 30 min of incubation with the air‐oxidized LTO1 at room temperature (22 °C).

LTO1 has recently been shown to interact with the lumenal segment of STN7 and oxidize the lumenal cysteines into an intermolecular disulfide bond *in vitro* [24]. To further validate this finding, we performed an *in vitro* redox reaction with MBP‐tagged LTO1 and the MBP‐tagged STN7 lumenal segment. We first fully reduced STN7 at room temperature for 30 min with 5 mm DTT, then removed the DTT by buffer exchange twice with a MiniTrap G25 column. The air‐oxidized LTO1 was then incubated with the reduced and buffer‐exchanged STN7 protein at room temperature (~ 22 °C). Figure 3 shows that the buffer‐exchanged STN7 protein migrates mostly as a monomer with a small amount of dimer (lane 1). LTO1 that has been air oxidized is also found in monomer and dimer forms (Fig. 3, lane 2). The abundance of the oxidized disulfide‐bonded dimeric STN7 was not increased upon incubation with LTO1 (Fig. 3, lane 3), contradicting the earlier finding by Wu *et al*. [24]. In the previous report, LTO1‐STN7 redox reaction was performed on ice rather than at ambient temperature as we have done. Further investigations are indeed necessary to uncover the basis of these differing observations. With our inability to resolve the intramolecularly disulfide‐bonded STN7 (Fig. 1), we cannot yet rule out the possibility that LTO1 has a role in catalyzing the formation of an intramolecular lumenal disulfide bond in STN7.

### Trx‐*f*1‐dependent reduction of stromal cysteines of STN7

**Fig. 4:**
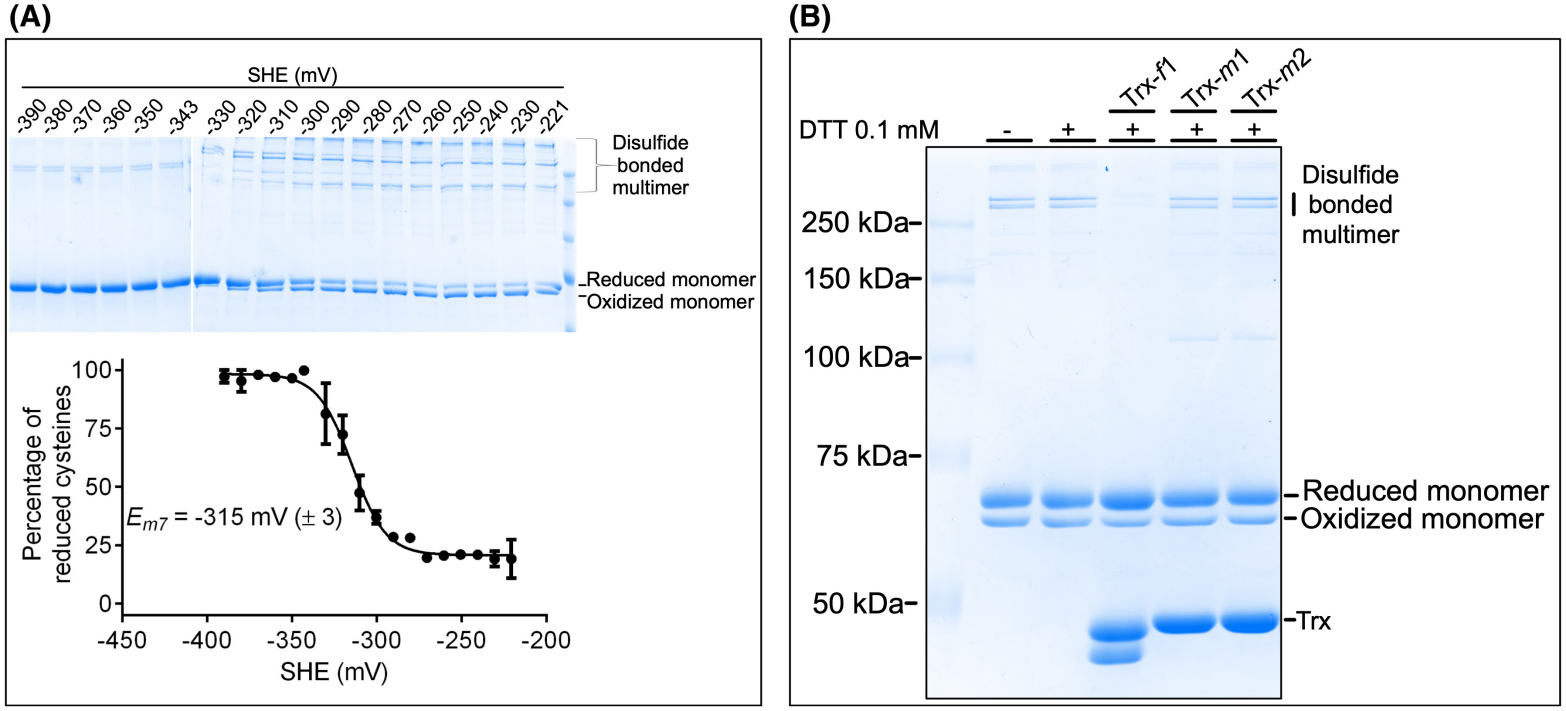
Redox properties of STN7 stromal cysteines. (A) Redox titration of the stromal cysteines of STN7. The nonreducing gel, containing the redox titration products, and the redox plot are shown. Error bars represent standard error of mean (SEM). (B) Trx‐*f*1 mediated reduction of the stromal disulfide linkage of STN7. Trx‐treated proteins as separated on nonreducing SDS‐urea‐PAGE gel and stained with Coomassie. + indicates the presence of 0.1 mm DTT.

The stromal cysteine motif has been suggested to be important for the inhibition of the plant STN7 by the ferredoxin–thioredoxin system [23, 26]. However, there have been conflicting reports on the ability of different thioredoxin isoforms to inhibit LHCII phosphorylation and to interact with STN7 [26, 27, 28]. We first determined the midpoint redox potential of STN7 stromal cysteines to see whether a reduction by thioredoxin is indeed feasible. As with the lumenal segment, we overexpressed and purified the stromal segment of STN7, containing the conserved C187 and C191 residues, as an N‐terminal fusion protein with MBP in *E*. *coli*. Unlike the lumenal segment, the monomeric oxidized (intramolecularly disulfide‐bonded) stromal segment migrates as a separate band on our nonreducing gel with no degradation products present (Fig. 4A). The monomeric reduced and disulfide bonded‐higher‐order oligomers of the stromal segment are also apparent on the gel. The redox titration reveals an *E*_m_ value at pH 7.0 of −315 ± 3 mV for the stromal cysteines (Fig. 4A and Data S1), as consistent with a reduction by thioredoxin. We subsequently carried out an *in vitro* Trx reduction assay to assess the ability of different thioredoxin isoforms to reduce the stromal cysteine disulfide bond of *Arabidopsis* STN7. We incubated the air‐oxidized protein with Trx‐*m* or Trx‐*f* in the presence of 0.1 mm DTT. Figure 4B shows the air‐oxidized stromal cysteine segment separated on nonreducing SDS‐urea‐PAGE gel as a reduced monomer, a low molecular weight oxidized monomer, and higher‐order oligomers. Incubation of the stromal segment with 0.1 mm DTT had no effect on the relative abundance of the oxidized monomer or higher‐order oligomers. Incubation of the stromal segment with Trx‐*f*1 in the presence of 0.1 mm DTT led to a complete disappearance of the higher‐order oligomers and a slight decrease in the oxidized monomer (Fig. 4B). A concomitant increase in the abundance of the reduced monomer is seen with Trx‐*f*1. In contrast to Trx‐*f*1, Trx‐*m*1 and Trx‐*m*2 had no effect on the relative abundance of the oxidized monomer or higher‐order oligomers.

## Discussion

**Fig. 5:**
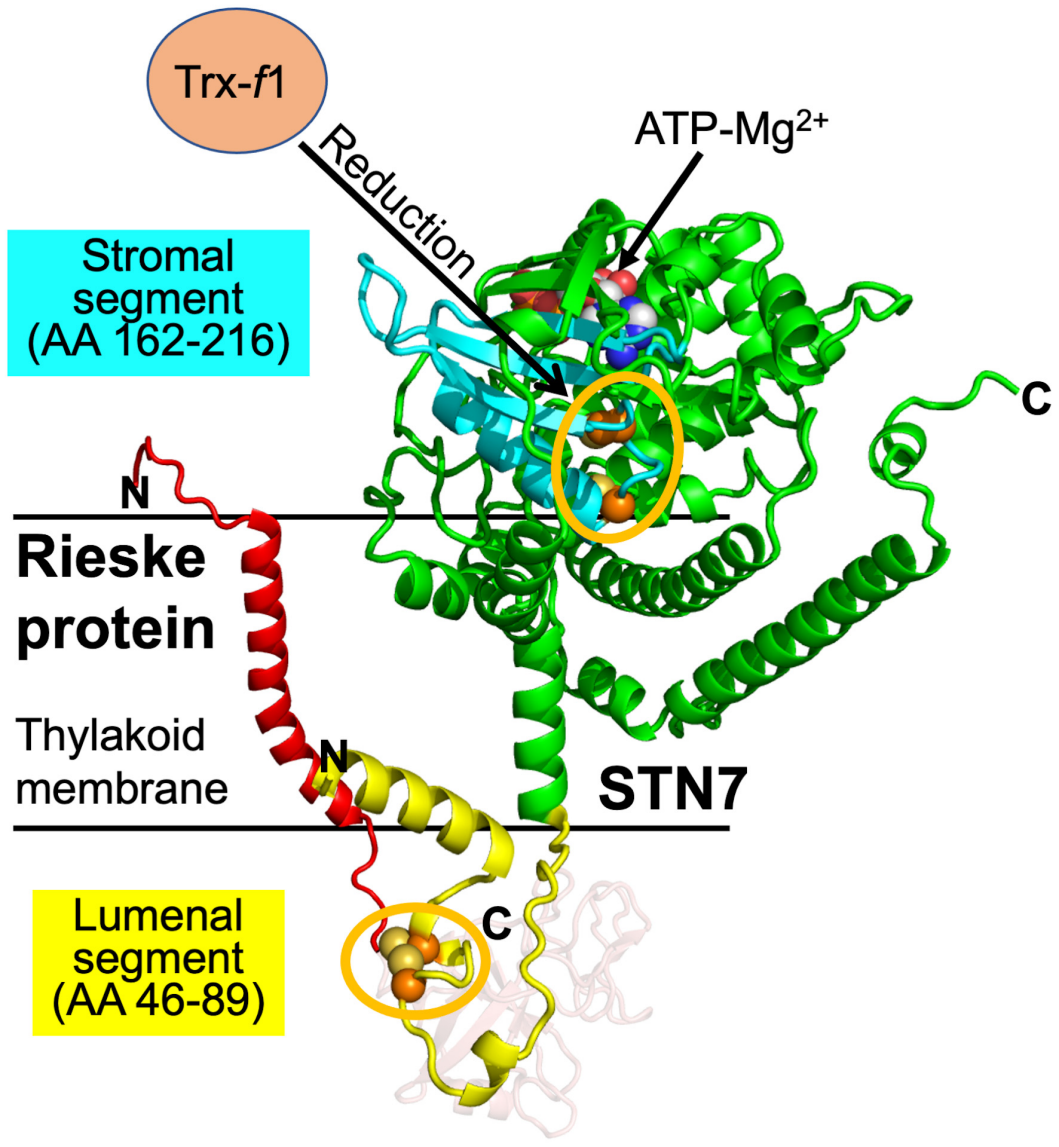
An AlphaFold 3‐predicted structure of mature *Arabidopsis* STN7 protein. The AlphaFold‐predicted STN7 structure has been manually placed alongside a cryo‐electron microscopy‐determined structure of plant Rieske Iron Sulfur Protein (6RQF, [37]). The STN7 amino acid regions corresponding to the recombinant lumenal and stromal segments used in this study are colored in yellow and cyan, respectively, and labeled. Likewise, the STN7‐interacting region of Rieske protein [16] is colored in red while Rieske's lumen‐located mobile head domain is shown in the background in semitransparent salmon color. The N‐ and C‐termini of both STN7 and Rieske protein are labeled. The side chains of the conserved lumenal and stromal cysteines of STN7 are shown as spheres and circled by orange ovals. A Mg^2+^‐ATP salt has been modeled into the kinase domain by AlphaFold 3. The lumenal location of the N‐terminal segment and the stromal orientation of the kinase domain of STN7 are based on Lemeille *et al*. [16]. The structure of mature STN7, spanning amino acids 45–563, was predicted using AlphaFold 3 [38].

Elucidation of the redox sensory mechanism of the LHCII kinase has remained a significant challenge given the intricate nature of its regulation. However, significant new insights have been obtained since the discovery of STT7/STN7 as the real LHCII kinase [6, 7]. A major finding is the identification of the conserved lumenal and stromal cysteines and their probable connection with the activation and repression of the kinase [6, 23] (Fig. 5). For the STT7/STN7 lumenal cysteines, a key question is whether they are subject to redox regulation such that these cysteines undergo a reversible disulfide to dithiol conversion as part of the activation mechanism. Our results do not favor this scenario (Figs 1, 2, 3). The large negative midpoint redox potential of the lumenal disulfide linkage(s) (*E*_m7_ ~ −300 mV, *n* = 2) makes its reduction by plastoquinol (*E*_m7_ +80, *n* = 2), plastosemiquinone (*E*_m7_ −154 mV, *n* = 1), superoxide (*E*_m7_ −140 mV, *n* = 1), or Rieske iron–sulfur protein (*E*_m7_ +320 mV, *n* = 1) thermodynamically unfavorable. We cannot however rule out the possibility of an as‐yet‐unknown reductant or an intermediate redox component between PQH_2_ and the lumenal disulfide of STN7. An earlier redox titration of thylakoid protein phosphorylation finds a midpoint redox potential of +38 mV (*n* = 1) for kinase activation while thylakoid protein phosphatase reactions were found to be redox‐independent [31, 32]. An *E*_m_ of +38 mV is indeed far apart from the highly negative *E*_m_ of the lumenal cysteines that we determine here but it is possible that the earlier study [31] titrated the *E*_m_ of a photosynthetic electron transport reaction that coincided with the kinase activation. A parsimonious explanation based on our data is that reversible disulfide to dithiol conversion of the lumenal cysteines does not occur during the activation of STT7/STN7. This is also consistent with the earlier observation that the lumenal disulfide linkage(s) of STT7 are not reduced in the timeframe of state transitions [17]. Some earlier studies discuss the functional kinase being dimeric as formed by intermolecular disulfide linkages between the lumenal cysteines [30]. However, this scenario is highly unlikely given our observations that the air‐oxidized lumenal segment forms very little dimers via intermolecular disulfide linkages (lane 2, Fig. 1A), even one of the strongest thiol oxidant, diamide, cannot fully convert all STN7 proteins into dimers (lane 5, Fig. 1A), and that STN7 dimerizes to a higher degree only when either of the two lumenal cysteines is removed. The kinase interacts with Rieske protein for its activation but the structural consideration that there is only one interaction surface of Rieske per the longer lateral face of the dimeric *b*_6_ *f* complex further makes functional dimer formation by STN7 tenuous. Admittedly, our and others' assumption on STT7/STN7 lumenal disulfide being an intramolecular linkage is based on circumstantial evidence such as PEGylation patterns and the absence of disulfide‐bonded intermolecular dimers *in vivo* [17, 30]. An alternate to intramolecular disulfide is that the sulfur atom of lumenal cysteines exist in other noncanonical oxidation states (sulfenic/sulfinic/sulfonic) as important for regulation of the kinase. However, the PEGylation patterns reported here and elsewhere [17] and the various observations on the regulatory properties of the LHCII kinase are inconsistent with such a notion [10, 20, 26]. Though we did not explicitly test here, a role for LTO1 in forming the lumenal intramolecular disulfide linkage of STT7/STN7 could still be assumed as consistent with LTO1's demonstrated function in catalyzing an intramolecular disulfide‐bond formation in the lumen‐resident PsbO protein of PSII [33]. Formation of disulfide bonds in the highly acidic thylakoid lumenal space may indeed require a catalyst like LTO1.

How would a rather inert intramolecular lumenal disulfide linkage facilitate STT7/STN7 activation? The tight folding of the lumenal segment, afforded by an intramolecular disulfide linkage, may facilitate the observed interaction between the kinase lumenal segment 44–91 and Rieske region 51–97 as necessary for kinase activation by a Rieske‐based conformational change mechanism [17] (Fig. 5). Indeed, the loss of either or both lumenal cysteines abolishes kinase activity [16]. From the AlphaFold‐predicted structure (Fig. 5), the N‐terminal region of STN7 appears to form a short membrane‐inserted alpha helix. This short helix may thus be available for interaction with the helical membrane anchor of Rieske protein. The adjacent disulfide linkage could permit proper folding of the STN7 N‐terminal short helix and or its interaction with Rieske transmembrane helix (Fig. 5). Under prolonged occupancy of the Q_o_‐site by a quinol (activation signal), such an interaction between Rieske and STN7 could be stabilized, shifting the kinase into an active state. Multiple proline residues are likely to introduce a kink in the transmembrane region of STT7/STN7 with unknown functional consequences [18] (Fig. 5). On the stromal side, STT7 interacts with the stromal loop residues of the cyt *b*_6_ *f* subunit IV, and this interaction is important for kinase activity [19]. Some versions of the conformational model for STT7/STN7 activation assume the cyt *b*_6_ *f*‐bound chlorophyll *a* molecule as the crucial redox sensor that transmits the activation signal to the kinase. The phytol tail of the chlorophyll *a* has been suggested to convey the occupancy of the Q_o_‐site to the kinase [21], and a latest model assumes volume changes in chlorophyll *a* molecule as relevant to the transmission of the activation signal across the thylakoid membrane [22]. With our results now disfavoring a thiol redox‐regulatory activation model of STT7/STN7, a more comprehensive testing of the nonredox‐based conformational change model should be forthcoming.

From studies of the high‐light induced inhibition of LHCII phosphorylation, a thioredoxin target site has been predicted to exist in the LHCII kinase [26]. This site was suggested to be dynamic as it becomes exposed only in high light (Fig. 5). The greater efficacy of more lipophilic and hydrophobic sulfhydryl‐directed reagents in inhibiting the LHCII kinase and the ability of ATP to preempt this inhibition have pointed to a buried thioredoxin target site presumably in the ATP‐binding domain [34]. The identification of such a CxxxC motif in the stromal ATP‐binding domain of STN7 fitted neatly with these early descriptions of a dynamic site [23] (Fig. 5). Our results suggest that the stromal cysteines form an intramolecular disulfide linkage, which could be reduced by DTT (Fig. 4A). The oxidized higher‐order oligomers are likely to be an artifact of the *in vitro* condition as seen also for the lumenal segment. The midpoint potential of the redox active stromal cysteines of STN7 at pH 7 is −315 mV (Fig. 4A) and plant Trx‐*f* and Trx‐*m* have midpoint potentials of −321 mV at pH 7.5 (*E*_m7_ −292 mV) and −335 mV (*E*_m7_ −306 mV) [35], respectively. The midpoint potential of Trx‐*f* and Trx‐*m* is thus closer to the *E*_m_ of stromal cysteines of STN7, suggesting that they may be ideal reductants of the stromal disulfide. Indeed, our *in vitro* thioredoxin reduction assay shows that Txr‐*f*1 reduces the stromal disulfide bond (Fig. 4B). Our results, together with the earlier observed interaction of STN7 specifically with Trx‐*f*1 rather than Trx‐*m* [27] and the greater efficiency of Trx‐*f* at inhibiting LHCII phosphorylation [26], suggest that STN7 is likely to be regulated by Trx‐*f*1 *in vivo* under high light [26, 27, 36]. It is then likely that the inhibition of LHCII phosphorylation by Trx‐*m* as noted by Ancín *et al*. in tobacco [28] is a nonspecific effect of Trx‐*m* overexpression. It is quite evident from the STN7 structure (Fig. 5) how a reduction of the stromal disulfide by Trx‐*f*1 could impair ATP‐binding and hence the kinase activity. Further biochemical and structural analyses of STN7 are indeed necessary to fully unravel the regulatory mechanism of this serine/threonine kinase that is central to photosynthetic acclimation.

## Author contributions

IMI, JRL, and SP conceived and conceptualized the study. IMI, JHL, SW, RK, and JRL devised methodologies, performed experiments, and curated data. IMI and SP wrote the manuscript with contributions from all coauthors. All authors have read and agreed to the published version of the manuscript.

### Peer review

The peer review history for this article is available at https://www.webofscience.com/api/gateway/wos/peer‐review/10.1002/1873‐3468.15032.

## Supporting information


**Data S1.** Redox titration datasets of STN7 lumenal and stromal cysteines.

## Data Availability

The dataset generated and analyzed in the current study is available as Data S1. All other data (if any) are available upon request.
