# Supplementary material for: Cysteine residues contribute to the regulation of Arabidopsis state transition 7 kinase
Source: FEBS Lett. 2024 Oct 11;599(3):436–46. doi: 10.1002/1873-3468.15032 (PMC11808413; doi:10.1002/1873-3468.15032)
Supplement: Supplementary file 1 — Data S1. Redox titration datasets of STN7 lumenal and stromal cysteines. [file FEB2-599-436-s001.zip › DataS1_Legend.docx]

**Supplementary Information**

**Supplementary Data 1. Redox titration datasets of STN7 lumenal and stromal cysteines**

Redox titration datasets of STN7 lumenal segment C1 and C2 mutant proteins at pH 7 and pH 8 and stromal segment protein at pH 7 are provided in an Excel table format. For DTT poised at different standard hydrogen electrode potential (SHE), the percentage of reduced cysteines is given. The latter was quantified from the intensity of the reduced protein band. Replicates refer to data from separate experiments.
